# Association between Gut Microbiota Composition and Copy Number Variations in Human Genes *FAM66D* and *TAS2R43*

**DOI:** 10.4014/jmb.2504.04011

**Published:** 2025-07-14

**Authors:** Jing Wang, Haoyu Guo, Weiwei Qi, Zhenyi Qiao

**Affiliations:** 1 Shanghai Key Laboratory of Bio-Energy Crops, School of Life Sciences, Shanghai University, Shanghai 200444, P.R. China; 2 State Key Laboratory of Dairy Biotechnology, Shanghai Engineering Research Center of Dairy Biotechnology, Dairy Research Institute, Bright Dairy & Food Co., Ltd., Shanghai 200436, P.R. China

**Keywords:** Gene copy number variations, gut microbiota, *FAM66D*, *TAS2R43*

## Abstract

The impact of the gut microbiota on human health has attracted increasing attention. However, the factors affecting the gut microbiota need more in-depth research. In this work, we analyzed the impact of the gut microbiota at the genetic levels, mainly gene copy number variations (CNVs). We used Permutational Multivariate Analysis of Variance (PERMANOVA, Adonis) analysis to assess the association between CNVs and the gut microbiota. The results showed that copy numbers of 50 genes varied among the cohort. Among them, the CNVs of 7 genes, including *TBC1D3L*, *OR4C6*, *NPIPB15*, *PDPR*, *USP17L7*, *FAM66D* and *TAS2R43*, were related to the gut microbiota. In addition, among these genes, we systematically analyzed the relationship between CNVs of *FAM66D* and *TAS2R43* with the gut microbiota. We hypothesize that there exists a certain association between genetic information in the human genome (such as CNVs) and human behavior as well as gut microbiota.

## Introduction

The impact of the gut microbiota on human health is being increasingly recognized. According to existing literature reports, the gut microbiota has extensive and finely tuned regulatory effects in intestinal inflammation [1, 2], tumor occurrence [3-5], Parkinson's disease [6-12], and autism spectrum disorder [13-15]. Corresponding to the human genome, the genome of the gut microbiota is known as the “second genome” of humans. However, the composition of this “second genome” is unstable compared with that of the human genome itself, the sequence of which exhibits long-term stability.

The unique genome of humans, like single-nucleotide polymorphism (SNP) and gene copy number variations (CNVs) of specific genes, is an important factor affecting the gut microbiota. Previous studies, by using microbial genome-wide association studies (mGWASs), have suggested that SNP sites on chromosomes are also considered to have the potential to regulate the gut microbiota [16-18]. For example, *Ruminococcus* and *Coprococcus* are associated with rs150018970 near *RAPGEF1* on chromosome 9, and rs561177583 within *LINC01787* on chromosome 1, respectively. In addition to SNPs, CNVs are also thought to be associated with the gut microbiota. Alpha-amylase 1 (*AMY1*) is a gene that encodes a salivary amylase [19]. The copy number of *AMY1* in the genome is closely related to obesity and insulin resistance [20-22]. People who have more *AMY1* copy sites in their genomes have a relatively unique gut microbiota. Mouse experiments have shown that this group of the gut microbiota can significantly increase the risk of obesity in normal mice [21]. In addition, due to the limitations of bioinformatics and the diversity of CNVs on chromosomes, there has still been no systematic analysis of the association of genome-wide CNVs with the gut microbiota.

In this study, we aimed to systematically reveal some of the associations among the genome and the gut microbiota through Permutational Multivariate Analysis of Variance (Adonis) analysis. We first analyzed the gut microbiota of 30 middle-aged women from Shanghai, China. In addition, we also performed whole-genome sequencing (WGS) of oral DNA and analyzed the results against the gut microbiota data. We found that 50 genes exhibited a difference in the number of copies of the complete coding region sequence. There was a association between the difference in the number of copies of 7 genes, such as *family with sequence similarity 66 member D* (*FAM66D*) and *taste 2 receptor member 43* (*TAS2R43*), and the change in the gut microbiota (*p* < 0.05). *TAS2R43* is a taste-related gene [23], and people with a high number of copies of this gene are more sensitive to caffeine [24]. Through an analysis of the gut microbiota and CNV data, we found that a high number of CNVs of *TAS2R43* resulted in changes in the gut microbial content that are partly related to health. This phenomenon may be the result of alterations in dietary habits caused by this gene.

## Materials and Methods

All experimental designs were approved by the Medical Ethics Committee of Shanghai Nutrition Society (19-SM-08-GM-002). WGS and 16S rRNA sequencing data have been uploaded to the National Omics Data Encyclopedia (NODE) (Project ID OEP001361 and OEP001366). The experiments involving fecal and oral samples were approved by the Ethics Committee of Zhongshan Hospital Affiliated with Fudan University.

### Sample Collection and Processing

After obtaining fresh stool samples, we immediately put them into a DNA preservation solution. Microbial DNA was extracted from fecal samples using the E.Z.N.A. Stool DNA Kit (Omega Bio-tek, Norcross, USA) according to the manufacturer’s protocols. The V3-V4 hypervariable regions of the bacterial 16S rRNA gene were amplified by a PCR thermocycler system (GeneAmp 9700, ABI, USA). The resulting PCR products were extracted from a 2% agarose gel, further purified using the AxyPrep DNA Gel Extraction Kit (Axygen Biosciences, USA) and quantified using the QuantiFluor-ST Fluorometer (Promega, USA) according to the manufacturer’s protocol. Oral swabs were used to collect the oral epithelial cells of the volunteers, and the cells were lysed and placed in a DNA preservation solution. Genome extraction was performed according to instructions using the TIANamp Swab DNA Kit (Tiangen Biotech, China).

### 16S rRNA Sequencing

A frozen aliquot (200 mg) of each fecal sample was suspended in 250 μl of guanidine thiocyanate, 0.1 M Tris (pH 7.5), and 40 μl of 10% N-lauroyl sarcosine. DNA was extracted using the Qiagen QIAamp DNA Stool Mini Kit (51504, Qiagen, Germany) according to the manufacturer’s instructions. The DNA concentration and molecular weight were estimated using a NanoDrop instrument (Thermo Fisher Scientific, USA) and agarose gel electrophoresis, respectively.

The V3-V4 region of the bacterial 16S ribosomal RNA gene was amplified by PCR (95°C for 2 min, followed by 25 cycles at 95°C for 30 sec, 55°C for 30 sec, 72°C for 30 sec, and a final extension at 72°C for 5 min) using the primers 338F 5'-ACTCCTACGGGAGGCAGCAG-3' and 806R 5'-GGACTACHVGGGTWTCTAAT-3', where the barcode was an eight-base sequence unique to each sample. PCRs were performed in triplicate in a 20 μl mixture containing 4 μl of 5 × FastPfu Buffer, 2 μl of 2.5 mM dNTPs, 0.8 μl of each primer (5 μM), 0.4 μl of FastPfu Polymerase, and 10 ng of template DNA.

Amplicons were extracted from 2% agarose gels and purified using the AxyPrep DNA Gel Extraction Kit (Axygen Biosciences, USA) according to the manufacturer’s instructions and quantified using the QuantiFluor-ST Fluorometer (Promega). Purified amplicons were pooled in equimolar amounts and paired-end sequenced (2 × 250) on an Illumina MiSeq platform according to standard protocols.

Raw fastq files were demultiplexed and quality-filtered using QIIME (version 1.9.1) with default parameters. Operational taxonomic units (OTUs) were clustered with a 97% similarity cutoff using UPARSE, and chimeric sequences were identified and removed using UCHIME. The taxonomy of each 16S rRNA gene sequence was analyzed by RDP Classifier against the Silva (SSU123)16S rRNA database using a confidence threshold of 70%. QIIME was used to calculate the diversity within communities (alpha diversity) and between communities (beta diversity). During the analysis of gut microbial community diversity, we used LEfSe and the Wilcoxon rank-sum test to identify differentially abundant microorganisms (LDA score > 2.0).

### Whole-Genome Sequencing

DNA was isolated using the DNeasy PowerClean Pro Cleanup Kit (Qiagen). A paired-end sequencing library with an insert size of 200 bp was constructed using the TruSeq DNA Sample Preparation Kit v2 (Illumina, USA) following the manufacturer's protocols. Purified libraries were quantified by a Qubit 2.0 Fluorometer (Life Technologies, USA) and validated by an Agilent 2100 Bioanalyzer (Agilent Technologies, USA) to confirm the insert size and calculate the molar concentration. Cluster generation was performed by cBot with the library diluted to 10 pM and then sequenced on the Illumina HiSeq 2500 (Illumina). Library construction and sequencing were performed at Sinotech Genomics Co., Ltd. (China).

### CNV Analysis

WGS was performed on 30 human oral samples. Library preparation and sequencing were performed at Sinotech Genomics. On average, 215.67 million high-quality reads were sequenced per sample, and 99.8% of the reads were aligned to a human reference genome (hg19). The average of all the samples’ mean depth was 8.67, and 82.7% of the whole genomes were covered, with a read depth > 5×.

Next, the germline copy number variant (gCNV) pipeline from the Genome Analysis Toolkit of the Broad Institute was used for copy number variant (CNV) detection [25]. GATK-gCNV uses PCA denoising and a panel of control samples to remove systematic sequencing noise, such as GC content, mappability, and other technical and systematic differences. GATK-gCNV also introduces a hierarchical hidden Markov model (HMM) for segmentation, which learns regions with common CNVs across multiple samples while simultaneously calling CNVs in each sample.

Specifically, fastp 0.20.1 was used to filter the raw reads, and BWA MEM 0.7.17 was used to align clean reads to hg19. GATK4 Mark Duplicates was used to mark duplicated reads. GATK4 Determine Germline Contig Ploidy was applied when modeling the background signals in the cohort samples. Then, GATK4 Germline CNV Caller was used to model the copy number signals, and the estimated copy number variation segments were extracted using GATK4 Post process Germline CNV Calls.

The CNVtools R package was used to estimate CNV activity in gene regions [26]. Then, dip.test from the R-diptest package was used to test the unimodality of the copy number distribution of these genes in the samples. Specifically, Hartigans' Dip statistics were calculated, and those genes with FDR < 0.05 were regarded as nonunimodal and were further analyzed to determine whether they interacted with microbes.

### qPCR

We determined the CNV of the *FAM66D* and *TAS2R43* genes by performing qPCR with an independent primer pair, *FAM66D*-forward: 5’-TCCAGGGAGGACCTGAGTTT-3’, *FAM66D* -reverse: 5’-GTCTCGGGCCTTGCAAAAAG-3’, *TAS2R43*-forward: 5’-TAATATCTGGGCAGTGATCAACC-3’, and *TAS2R43*-reverse: 5’-CCCAACAAC ATCACCAGAATGA-3’. We also used the aforementioned primers for *EIF2B2* as a reference gene on the same qPCR plate. Primers were at 0.5 mM, and 2 ng of DNA template per reaction were used in 20 μl reactions with Thermo Fisher Quantstudio 3. *EIF2B2* was run on the same plate with both *FAM66D* and *TAS2R43*. Reactions were performed in triplicate.

## Results

### Participant Recruitment and Screening

**Fig. 1:**
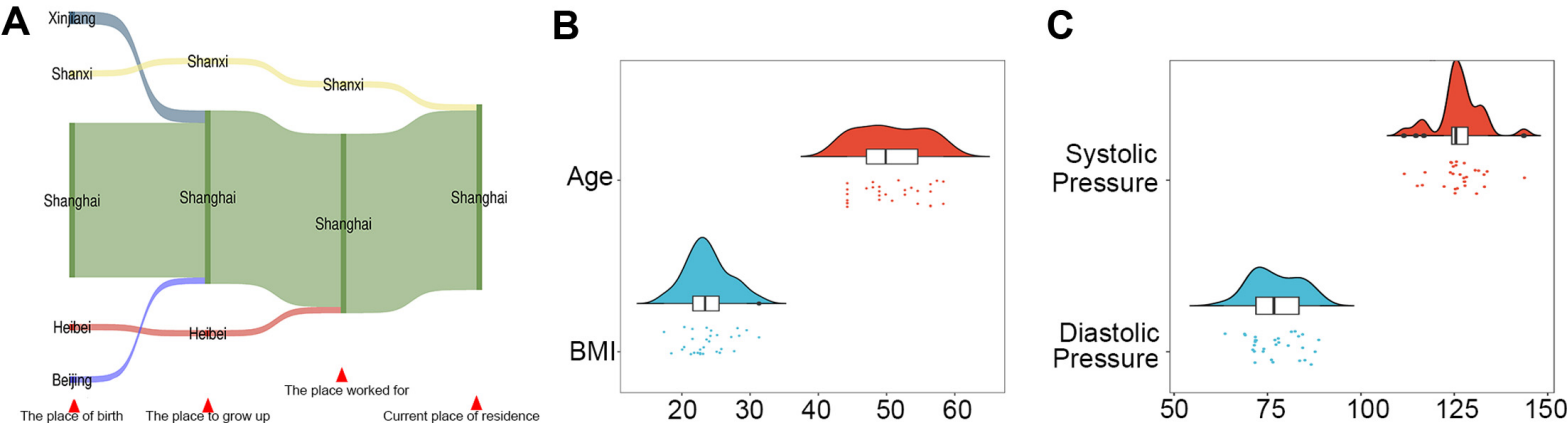
Demographic and biometric characteristics of the cohort. (**A**) Sankey diagram illustrating mobility patterns of 30 volunteers. (**B**) Statistical analysis of BMI-age distributions. (**C**) Systolic/diastolic blood pressure profile analysis.

To minimize potential confounding effects from regional lifestyle variations and ethnic genetic heterogeneity, we exclusively recruited same-sex Han Chinese adults with long-term residency in the same geographical area as genetic material sources, ensuring a homogeneous cohort for experimental controls. We recruited 30 volunteers from the general population in Shanghai, China. Although these volunteers had different places of birth and workplaces, they had been living in Shanghai for a long time (more than five years) (Fig. 1A). The female participants, aged 45-60 years (mean ± SD: 52 ± 5.13), maintained body mass index (BMI) and blood pressure values within established clinical reference ranges (Fig. 1B and 1C). In addition, we excluded volunteers who had severe digestive tract diseases and other major illness, such as intestinal tumors and cardiovascular disease, and those who had undergone cholecystectomy. Volunteers who had consumed antibiotics or had other behaviors that could have impacted the gut microbiota in the previous three months were also excluded from the cohort. In addition, volunteers with unclear consciousness and mental illness were also excluded. Although we cannot guarantee that these standards completely excluded the sick population, considering that our purpose was to widely screen CNVs affecting the gut microbiota, we believe that these standards were enough for us to obtain high-quality population samples. Based on these criteria, we recruited 30 volunteers, collected fecal samples for 16S rRNA sequencing, and administered behavioral questionnaires. The questionnaires asked about social characteristics, health status, smoking, alcohol consumption, diet, exercise, sleep, cosmetic use, and raising animals and plants. This enabled us to make a more comprehensive judgment about whether our volunteers were in good health.

### Gut Microbiota Profiling of the Cohort

**Fig. 2:**
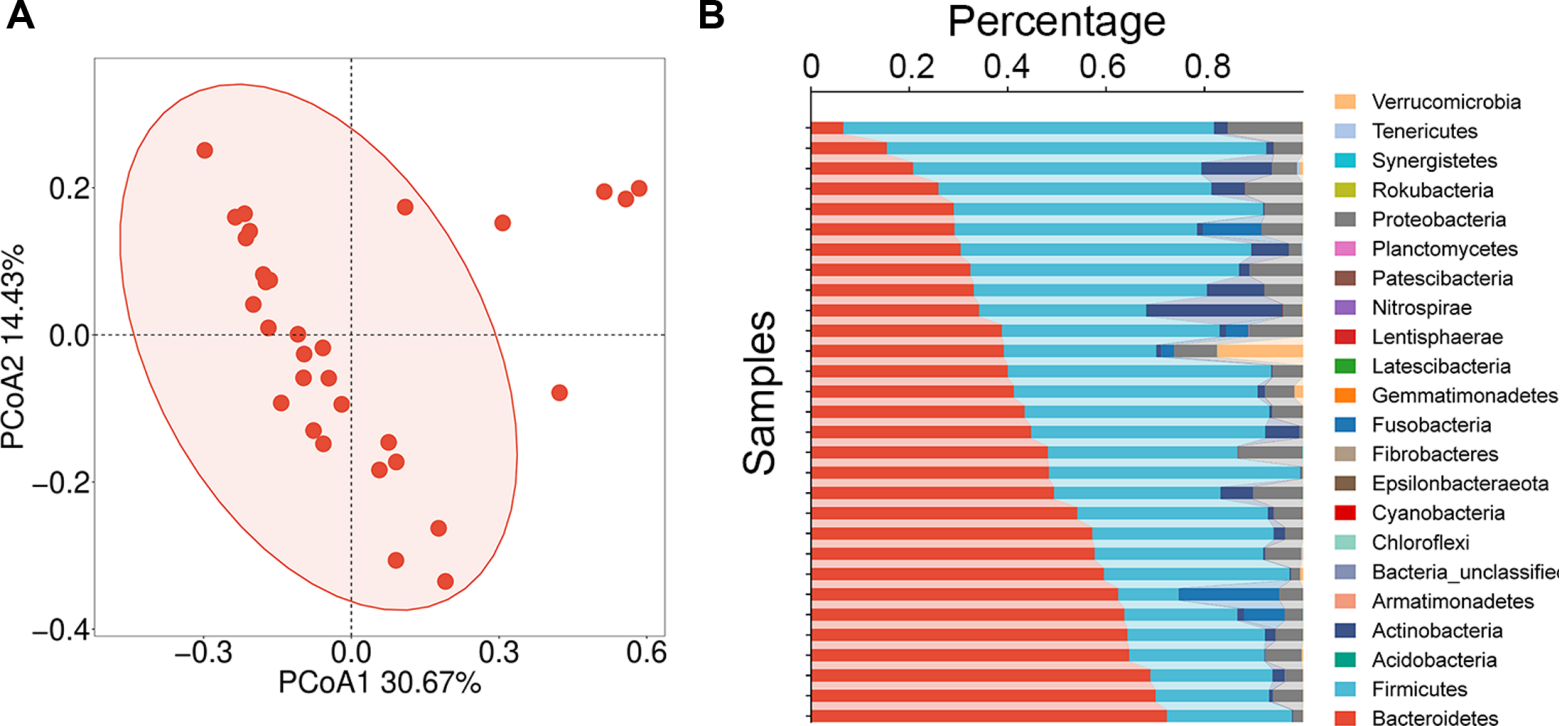
Gut microbiota composition of the cohort. (**A**) PCoA plot with 95% confidence ellipse. The arrows represent the contribution of the composition of microbiota to the principal components. (**B**) Phylum-level taxonomic composition (color-coded by distinct phylogenetic groups).

Principal Coordinate Analysis (PCoA) is a multivariate statistical method used to visualize and explore similarities or dissimilarities between samples based on a distance matrix. It is widely applied in ecology, genomics, and microbiome studies to reduce the dimensionality of complex datasets while preserving the original distance relationships. Initial analysis of fecal samples from 30 volunteers using PCoA revealed clustering patterns where most samples aggregated within the central ellipse, while outliers deviated from this core distribution (Fig. 2A). This intrinsic heterogeneity within the cohort, while preserving dominant gut microbial signatures, allowed targeted investigation of strains exhibiting association with CNVs. Taxonomic profiling showed Bacteroidetes and Firmicutes as the most abundant phyla (Fig. 2B). However, considerable inter-individual variability in their relative abundance was observed. Beyond these dominant taxa, Proteobacteria and Actinobacteria showed detectable enrichment across subsets of the cohort.

**Fifty genes with different genomic copy numbers were screened using genome-wide CNV analysis.**

**Fig. 3:**
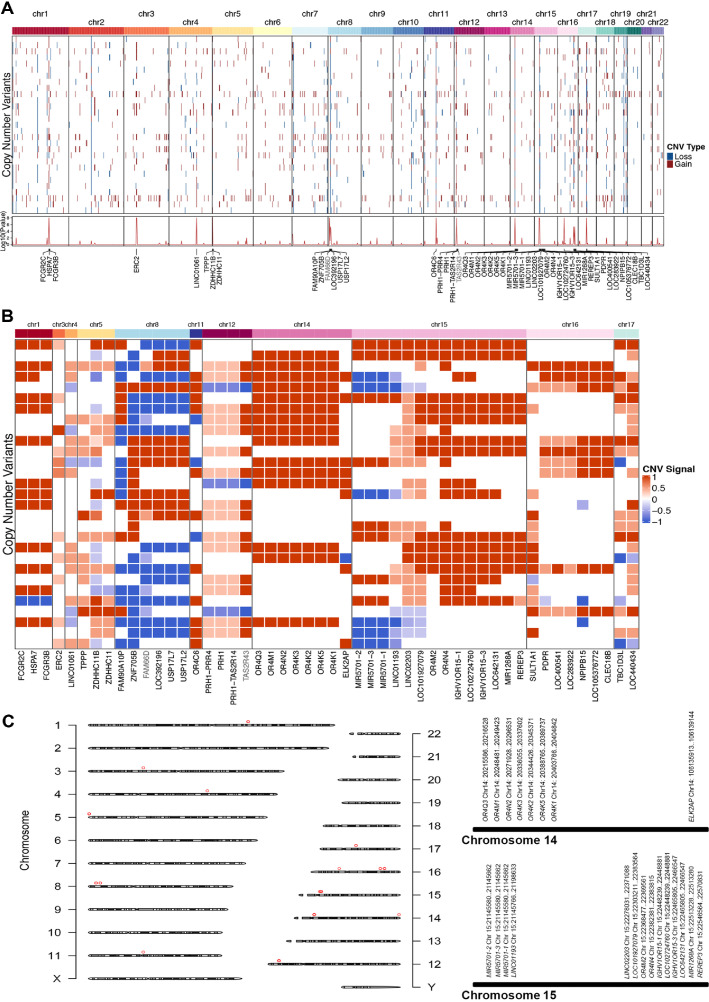
Genome-wide CNV analysis of 29 samples. (**A**) Twenty-nine samples with CNVs in different chromosomes. Red represents segments with increased copy number (gain), and blue represents segments with decreased copy number (loss). (**B**) The heatmap shows 29 samples with CNVs in different chromosomes. Red represents fragments with increased copy number, and blue represents fragments with decreased copy number. The CNV signal range reflects the confidence level for each gene identified with copy number variations across individuals. Values closer to 1 indicate high copy numbers with greater confidence, while values closer to -1 indicate low copy numbers with higher confidence. (**C**) Chromosomal architecture and CNV localization. Schematic representation of chromosome structural organization. Genomic coordinates of chromosomal regions harboring CNVs, with red open circles demarcating loci exhibiting statistically validated CNVs.

To explore the regulatory effect of CNVs on the gut microbiota, we obtained oral genome samples, and performed WGS. Genes have many types of CNVs in chromosomes, such as uniparental disomy (UPD) and loss of heterozygosity (LOH). Here, we counted the number of copies according to the chromosome section (Supplementary Fig. S1). If the number of copies changed in a section of the chromosome, the genes in this segment were counted as increased or missing (*q*-value < 0.05). Among the 30 samples, we found that one had a wide range of gains. Considering the possibility of experimental error, we excluded this sample. In addition, we excluded genes that had the same copy numbers in the genomes of the 29 volunteers. Although CNVs were detectable across all chromosomes (Fig. 3A), statistical analyses identified 50 genes on 11 chromosomes exhibiting significant CNV alterations (FDR < 0.05) (Fig. 3B, Table S1). Notably, multiple genes on chromosome 14 (Chr14: 20336055-20215586) and chromosome 15 (Chr15: 21145662-22371088) exhibited marked CNV differences (Fig. 3A and 3C), suggesting potential large-scale chromosomal variations in these genomic regions. Therefore, we used WGS data from 29 samples for the subsequent genomic analysis. Furthermore, we performed association analysis with the gut microbiota of the 50 genes described above by the Adonis test (Table S1). Adonis analysis is a non-parametric statistical method based on distance matrices. It is commonly used in fields such as ecology, microbiome research, and related disciplines to test for significant differences in multivariate data (*e.g.*, species composition, CNVs) between groups. Based on this analysis, seven genes, namely *TBC1D3L*, *OR4C6*, *NPIPB15*, *PDPR*, *TAS2R43*, *USP17L7* and *FAM66D*, were correlated with changes in the gut microbiota (p-value < 0.05) (Table 1). Among these seven genes, *FAM66D* is a long, non-coding RNA (lncRNA) considered to play a role in inflammatory bowel disease (IBD) and tumor development, while *TAS2R43* is a gene associated with taste. Considering that IBD and taste are closely related to the gut microbiota, we conducted an in-depth study on these two genes.

CNV of *FAM66D*, a lncRNA that affects the composition of the gut microbiota.

**Fig. 4:**
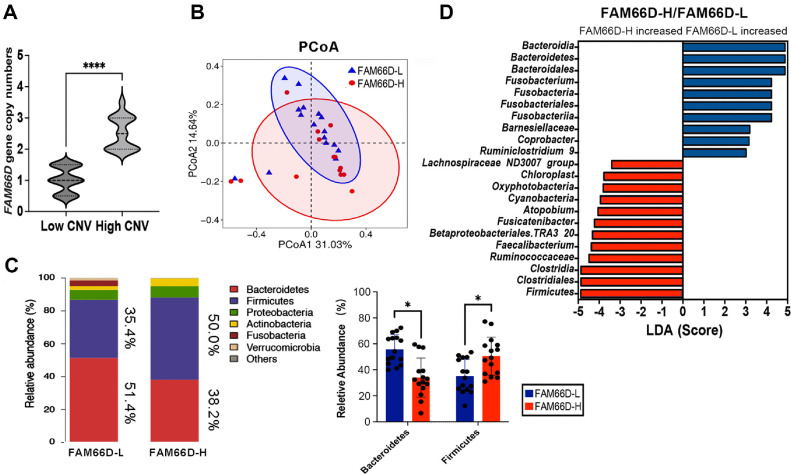
Association analysis of *FAM66D* gene CNV and intestinal microorganisms. (**A**) Statistical analysis of high CNV and low CNV of *FAM66D* (****: *p* < 0.0001, Student’s t-test). (**B**) PCoA was used to analyze the differences in intestinal microorganisms between the FAM66D-L and FAM66D-H groups. (**C**) The proportions of Bacteroidetes and Firmicutes in the FAM66D-L and FAM66D-H groups. (**D**) Linear discriminant analysis (LDA) identifies the abundance of intestinal microorganisms in the FAM66D-L and FAM66D-H groups.

*FAM66D* (ENSG00000255052) is a gene located in chromosome 8. According to the annotation of the human GRCh38.p14 reference genome, its specific location is chr8:12,115,767-12,202,753. *FAM66D* was previously considered to be a lncRNA involved in IBD and tumor growth [27, 28]. In this study, we found that the copy numbers of the *FAM66D* gene were different in 29 sample genomes (*p* < 0.001) (Fig. 4A). We validated the copy number variation of *FAM66D* in the cohort using qPCR. The samples with high copy numbers (FAM66D-H) and low copy numbers (FAM66D-L) were divided into two groups. Through genome sequencing coverage analysis, we found that the coverage of reads in the FAM66D-H group was higher than that of reads in the FAM66D-L group (Fig. S2). The PCoA analysis results of gut microorganisms with 16S rRNA sequencing showed that the gut microbiota composition of FAM66D-L and FAM66D-H exhibited partial differences (Fig. 4B). Interestingly, at the phylum level, the Firmicutes content in the FAM66D-H group (50.0%) was higher than that in the FAM66D-L group (35.4%) (Fig. 4C). In contrast, the Bacteroidetes content decreased in FAM66D-H (38.2%) (Fig. 4C). Considering that the Firmicutes/Bacteroidetes ratio often represents the metabolic capacity of the host, we believe that the CNV of *FAM66D* may be related not only to IBD and tumors, but also to metabolism. However, more investigation is needed to confirm the specific molecular mechanism. Furthermore, we also found that *Fusobacterium* decreased significantly in FAM66D-H (Fig. 4D). Considering the positive correlation between *Fusobacterium* and cancer, we believe that people with a high CNV of *FAM66D* may be associated with cancer and intestinal inflammatory disease.

***TAS2R43*, a taste-related gene, is associated with changes in dietary habits, and thus affects the composition of the gut microbiota.**

**Fig. 5:**
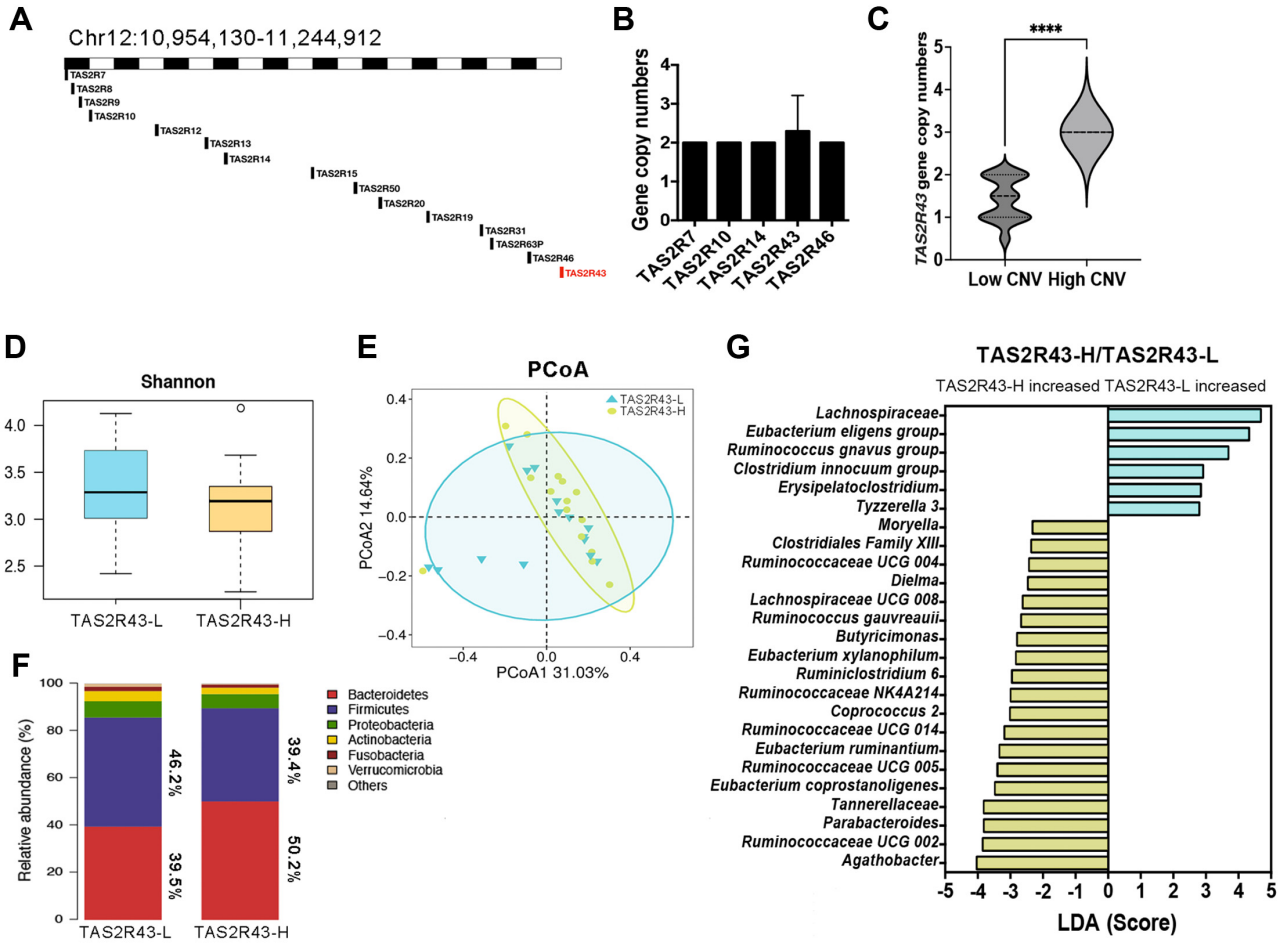
Analysis of the associations among behavior, the gut microbiota, and *TAS2R43* CNV. (**A**) Diagram of the *TAS2R43* gene locus on the chromosome. The *TAS2R43* gene is highlighted in red. (**B**) CNV analysis of 5 genes related to taste. Error bars represent ± SD. (**C**) Statistical analysis of high CNV and low CNV of *TAS2R43* (****: *p* < 0.0001, Student’s t-test). Error bars represent ± SD. (**D**) Shannon index of the gut microbiota of the TAS2R43-L and TAS2R43-H groups. (**E**) PCoA was used to analyze the difference in the gut microbiota between the TAS2R43-L and TAS2R43-H groups. (**F**) The proportions of Bacteroidetes and Firmicutes in the TAS2R43-L and TAS2R43-H groups. (**G**) LDA identifies the abundance of intestinal microorganisms in the TAS2R43-L and TAS2R43-H groups.

*TAS2R43* (ENSG00000282537) is a taste-related gene [23]. People with high CNV in this gene are more sensitive to caffeine, which makes them less able to tolerate bitter tastes. In chromosome 12, there were 15 members of the TAS2 family (Fig. 5A). Among them, *TAS2R7*, *TAS2R10*, *TAS2R14*, *TAS2R43*, and *TAS2R46* are considered to be related to taste. We counted the CNVs of the five genes and found that only *TAS2R43* had CNVs (0-3 copies) in our cohort (Fig. 5B). Therefore, we divided the 15 people (0-2 copies) with low CNV (TAS2R43-L) and 14 people (3 copies) with high CNV (TAS2R43-H) into two groups. Through genome sequencing coverage analysis, we found that the coverage of reads in the TAS2R43-H group was higher than that of reads in the TAS2R43-L group (Fig. S3). According to these results, we further investigated the association between *TAS2R43* and the gut microbiota (Fig. 5C). We also validated the CNV of *TAS2R43* in the cohort using qPCR.

First, gut microbiota composition data indicated that the species diversity of the TAS2R43-L group was higher than that of the TAS2R43-H group (Fig. 5D). PCoA analysis of intestinal microorganisms with 16S rRNA sequencing showed that the gut microbiota composition of the TAS2R43-L and TAS2R43-H groups was also different (Fig. 5E). The Firmicutes/Bacteroidetes ratio (0.785) in the TAS2R43-H group was lower than that in the TAS2R43-L group (1.170) (Fig. 5F). This suggests that the TAS2R43-H group may have had a higher energy metabolic efficiency. Moreover, we found changes in the levels of 25 intestinal microorganisms, including *Agathobacter*, the *Eubacterium eligens* and *Ruminococcus gnavus* groups, *Butyricimonas*, *Erysipelatoclostridium*, and *Coprococcus 2* (Fig. 5G). Considering that *TAS2R43* is a taste-related gene, this suggests that *TAS2R43* may regulate the composition of gut microbiota through changes in dietary habits.

## Discussion

There are obviously more factors affecting genomic changes in the gut microbiota than those in the human genome. Although there is consensus that human genomes influence the composition of the gut microbiota, previous studies have focused more on the effects of single genomic loci (CNVs and SNPs) or single disease phenotypes on the gut microbiota [16-18, 21]. Systematic studies of how the genome information affects the gut microbiota remain scarce. Here, we screened 29 volunteers to correlate their genetic hallmarks with their gut microbiota. Among them, 30 samples were selected for association analysis of CNVs.

Although many studies have reported that partial variations in human chromosomes are correlated with the gut microbiota and induce disease through the gut microbiota, these studies are limited to mGWASs of only some diseases [16-18]. Genome-wide screening for genetic traits that affect the gut microbiota are rarely performed. Here, we first carried out WGS on oral genome samples of 30 volunteers and analyzed the CNVs of all the genes. After associating CNV data with the gut microbiota, we screened genes that could regulate gut microbiota composition, such as *FAM66D* and *TAS2R43*. It has been previously reported that *FAM66D* is a lncRNA that plays a promoting role in IBD and the development of cancer [28]. However, until now, there have been no reports indicating that *FAM66D* could affect the composition of the gut microbiota members, such as *Fusobacterium*. Here, we not only screened CNVs affecting the gut microbiota for the first time on a genome-wide scale, but also explored the influence of lncRNAs that play a role in IBD and the development of cancer on the gut microbiota. Since *Fusobacterium* has been shown to promote colorectal cancer (CRC) [3], we believe that the CNV of *FAM66D* in the genome can also be used as a risk assessment indicator for CRC. In addition, *TAS2R43* is a gene associated with taste. We hypothesize that there exists a certain association between genetic information in the human genome (such as CNVs) and human behavior as well as the gut microbiota.

With widespread awareness of precision medicine and precision nutrition around the world, an increasing number of national governments, universities, hospitals, biological enterprises, pharmaceutical enterprises and food enterprises have invested a wealth of resources in intestinal health research. However, current approaches to improving health and alleviating disease by altering the gut microbiota, such as fecal microbiota transplantation (FMT) and probiotic use, are highly uncertain due to the many factors that influence the gut microbiota. This is not conducive to the popularization of precision nutrition. We believe that it is necessary to fully consider the genetic attributes and behaviors of the adaptive population in gut microbiota research and technology development. With this in mind, we believe that our work will promote the industrial application of the gut microbiota.

However, our work also has great limitations. Due to the small sample size, we studied CNVs in the genome but did not conduct in-depth analysis of SNPs through mGWAS. In addition, our volunteers were limited to women between the ages of 45 and 60, so our results may not be universal for all genders, races, and ages. Finally, although we found that many gut microbes are influenced by genetic attributes, the functions of these gut microbes are still unknown. Therefore, further research is needed to confirm the biological functions of these microbes.

## Supplemental Materials

Supplementary data for this paper are available on-line only at http://jmb.or.kr.



## Figures and Tables

**Table 1:** List of genes that affect the gut microbiota.

Gene name	Description	Chr	Pr(>F)
*TBC1D3L*	TBC1 domain family member 3L	17:37978155-37989060:-1	0.015
*OR4C6*	Olfactory receptor family 4 subfamily C member 6	11:55662201-55666195:1	0.023
*NPIPB15*	Nuclear pore complex interacting protein family member B15	16:74376306-74392115:1	0.025
*PDPR*	Pyruvate dehydrogenase phosphatase regulatory subunit	16:70114332-70162537:1	0.031
*TAS2R43*	Taste 2 receptor member 43	12:11091287-11092313:-1	0.032
*USP17L7*	Ubiquitin specific peptidase 17 like family member 7	8:12132417-12134099:-1	0.046
*FAM66D*	Family with sequence similarity 66 member D	8:12115767-12202753:1	0.047
