# Supplementary material for: Association between Gut Microbiota Composition and Copy Number Variations in Human Genes FAM66D and TAS2R43
Source: J Microbiol Biotechnol. 2025 Jul 14;35:e2504011. doi: 10.4014/jmb.2504.04011 (PMC12283257; doi:10.4014/jmb.2504.04011)
Supplement: Supplementary file 1 [file jmb-35-e2504011-supple.pdf]

J. Microbiol. Biotechnol. 2025. 35: e2504011

https://doi.org/10.4014/jmb.2504.04011

Supplementary Figures

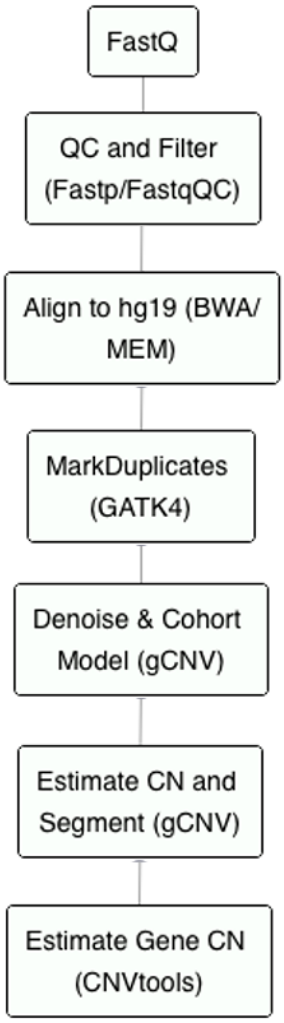

Fig. S1. CNV analysis process diagram.

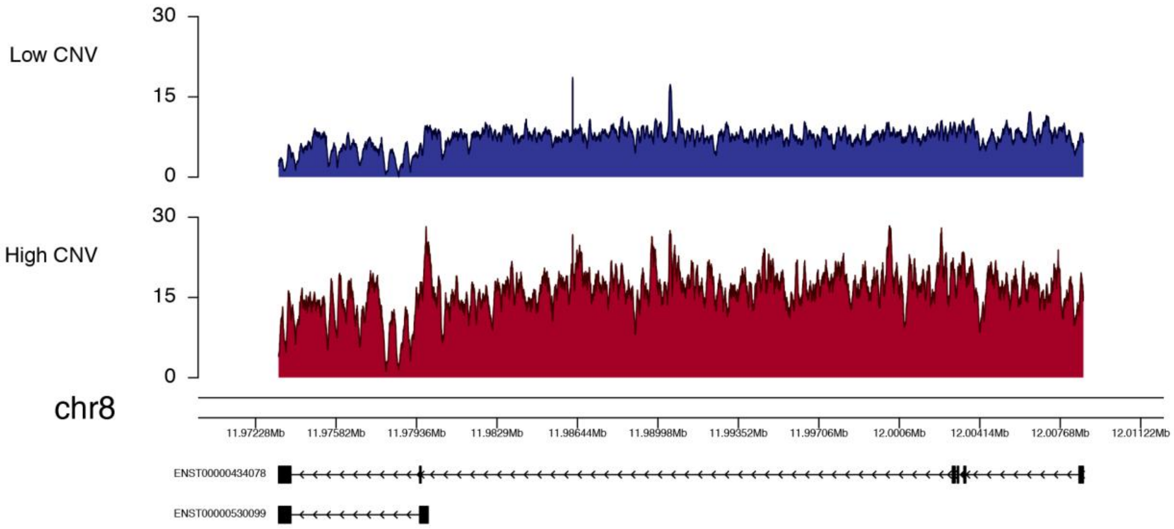

Fig. S2. Average read coverage in *FAM66D*.

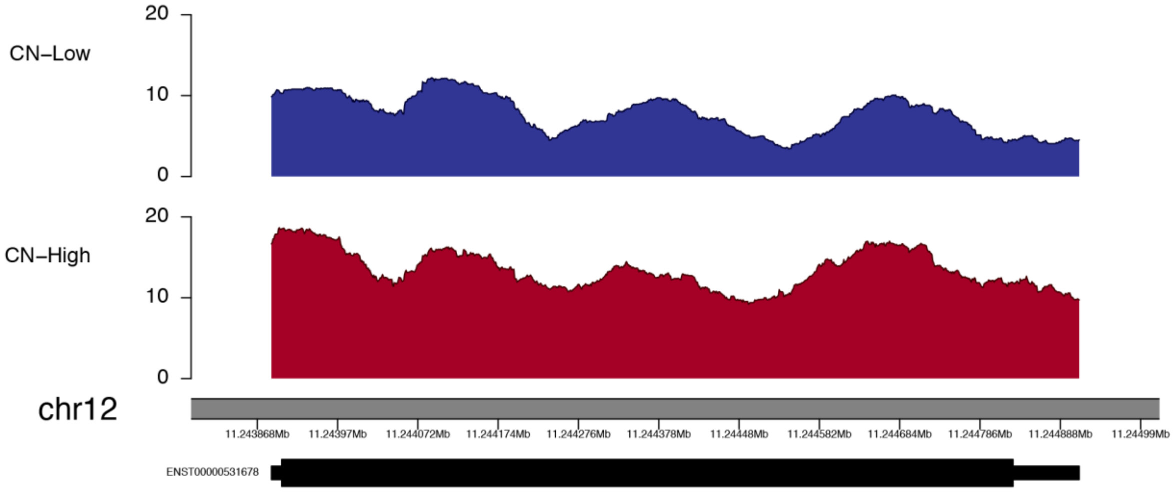

Fig. S3. Average read coverage in *TAS2R43*.
